# Autofluorescence Images with Carl Zeiss versus Topcon Eye Fundus Camera: A Comparative Study

**DOI:** 10.1155/2013/309192

**Published:** 2013-04-22

**Authors:** Juan M. Muñoz, Rosa M. Coco, M. Rosa Sanabria, Ruben Cuadrado, Eduardo Blanco

**Affiliations:** ^1^Instituto de Oftalmobiologia Aplicada (IOBA), Universidad de Valladolid, Spain; ^2^Consultorio 225, Consultorios América, Vía España, Panamá, Panama; ^3^Complejo Asistencial de Palencia, Spain

## Abstract

*Purpose*. To compare the autofluorescence images of the Zeiss versus Topcon eye fundus cameras and design an objective way to quantify it. *Procedures*. The IMAGEJ software was used to determine the gray level corresponding to the darkest veins and the peripapillary ring (thresholds), the level of white of the brightest perifoveal area, their difference (contrast level), and the suprathreshold area for each photograph. *Results*. Carl Zeiss has higher contrast values than Topcon. The Topcon contrast presented a crest with further decline as the suprathreshold area continued to increase. On the contrary, the Zeiss profile did not decline in contrast. *Conclusions and Message*. The Carl Zeiss camera showed superior contrast ability over the Topcon when performing autofluorescence imaging. We set objective parameters to compare fundus cameras FAF images. These parameters could be the base to objectively measure and determine changes and realize followup to areas of hyper- or hypofluorescence.

## 1. Introduction

Fundus autofluorescence (FAF) imaging is a noninvasive imaging method which provides additional information not obtainable with other imaging techniques [1], or ordinary fundus examination [2]. FAF became recently available in the last decade with the introduction of confocal laser scanning ophthalmoscopes, by using an exciting wavelength of 488 nm and detecting the light emitted above 500 nm [3].

There are several tissues having autofluorescent properties within the eye, such as the cornea, the lens, the retinal pigment epithelium (RPE), the uveal melanocytes, and the scleral collagen, but the main source of fundus autofluorescence is lipofuscin localized at the level of the RPE [4, 5]. Lipofuscin is intrinsic retinoid fluorophores, toxic coproduct of the photoreceptors, that accumulates in the lysosomes of the RPE, possibly due to incomplete degradation of photoreceptor outer segments. The concept of FAF relies on natural fluorescence occurring from the retinal layers, providing an indicator of the RPE monolayer health. FAF imaging may identify first signs of retinal diseases before they are evident. Low pixel values (dark) illustrate low intensities and high pixel values (bright) high intensities, respectively. The optic nerve head and retinal vessels typically appear dark as they obscure the normal RPE FAF underlying [6]. Besides the intensity of FAF (log⁡[*F*(end)]) is lowest at the center of the fovea and increased gradually toward the periphery within a 270-pixel square [7]. Moreover, FAF can be correlated with specific patterns identified by autofluorescence and can also be used as a monitoring tool after therapeutic procedures as retinal detachment, macular surgery, or laser treatment [3, 8–11].

The available data on FAF imaging suggest that it is possible to relatively differentiate between normal and abnormal FAF intensities over certain regions of the retina in individual patients. However, little has been published regarding FAF imaging methods able to objectively quantify alterations in the magnitude and localization of the FAF intensity in a single patient or between patients [1, 8]. In addition, although the photopigment has been assessed in Scanning Laser Ophthalmoscopy (SLO) pictures by using the fluorescence optical density difference (fODD) [12], no quantitative models have been described, to quantify and compare autofluorescence images from eye fundus cameras. Even though the images of SLO are always focused and seem to provide the clinician a better resolution, eye fundus cameras are more widespread and available in many ophthalmologic centers.

Finally, the contrast might be considered as the main indicator of quality of a gray photograph and refers to the ability to capture dark and bright zones at the same time [13]. It depends on multiple factors including reflectivity of the optic system and the illuminance [14]. The purpose of our study was to compare the quality of contrast imaging FAF between the Zeiss and Topcon camera. In order to do that, we also developed a quantitative method to compare the FAF by comparing the contrast levels of the two images, determining areas to be used as reference and thresholds, calculating suprathreshold areas of both cameras, and establishing a correlation between contrast and suprathreshold areas.

## 2. Methods

In our study, we took FAF photographs to every patient sent to the IOBA (a referral center) from the CAPA, who fulfilled the following criteria: (1) eyes without retinal detachment, (2) transparent media (cornea, anterior chamber, lens, and vitreous), (3) good cooperation to allow the capture of the photographs, and (4) proper health condition to allow the photograph capture.

We compared the Topcon TRC-50IX with a Kodak Megaplus 1.4i camera and the Zeiss FF 450 Plus IR with Visupac 4.1, and a Kodak Megaplus 1.6 camera (available in IOBA and CAPA, resp.). The Carl Zeiss used an exciter filter of 510–580 nm and a barrier filter of 650–735 nm, while the Topcon used an exciter filter of 500 to 610 nm and a barrier filter of 675 to 715 nm. All the photographs were taken by certified eye fundus photographers, including 50° of the posterior pole, centered on the fovea.

We used the IMAGEJ software from the National Health Institute of the United States, specially designed for images analysis. The software is able to determine the gray level of any point within the picture and to locate it in a scale of 256 gray levels, where 0 is absolute black, and 255 is absolute white. The software is also able to calculate a suprathreshold area over a gray level within a photograph. The images were brought to the same size for the comparison. All the 50^a^  images were resized 1280 × 1024 pixels before being processed.

To determine the contrast (what we called “Δ”), we measured the gray level of 3 points within the darkest peripapillary ring, 3 points in the darkest areas of the biggest veins, and 3 points in the brightest perifoveal area. For each photograph, we estimated 2 “Δ” values. The first one was the difference between the average of the brightest perifoveal points and the average of the darkest points of the veins. The second was the difference between the average of the brightest perifoveal points and the average of the darkest peripapillary points. When comparing the Δ values between different areas, we used the formula: $t=\overset{-}{x}T-\overset{-}{x}C/\text{sq}\left(\text{var}T/nT+\text{var}C/nC\right)$ where alfa = 0.05, and degrees of freedom = 24. We also calculated the brighter area of the photographs over the corresponding average of the veins gray level and over the corresponding average of the peripapillary ring area.

For the statistical processing, we performed the Student's *t*-test for two independent samples assuming unequal variances. We used the Microsoft Excel to calculate regression models to find out any correlation between the contrast levels and the suprathreshold areas in the photographs.

The informed consent was obtained. The researchers have no commercial interests in this study. This study followed the tenets of Helsinki.

## 3. Results

Thirteen autofluorescence images from seven patients were included. One eye of one of the patients was excluded because of media opacity.

When comparing the  Δ values between veins and the perifoveal area using the formula given above, we obtained a *t* = 2.86, for a *P*  value = 0.006, indicating statistical differences, with higher contrast values for the Carl Zeiss camera. When comparing the Δ values between the peripapillary ring and the perifoveal area, we found that *t* = 3.44, for *P*  value = 0.001, indicating a significant difference and a higher contrast for the Carl Zeiss camera too.

**Figure 1:**
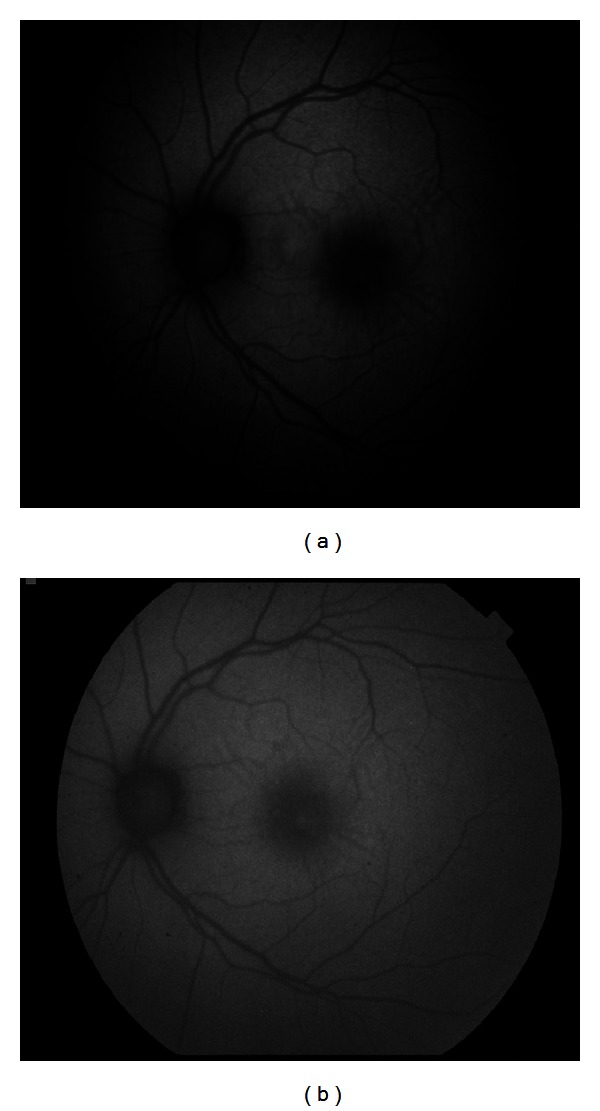
Macular star photographed with the Carl Zeiss camera (a) and the Topcon camera (b). Notice more marked difference between light gray and dark gray for the Carl Zeiss and the better defined mask for the Topcon.

For the visible area over the veins threshold, we found no difference between the two cameras (*t* = 0.759, *P* value = 0.23), but we did find differences for the visible area over the peripapillary ring threshold between the two cameras (*t* = 2.60, *P* = 0.01). The largest suprathreshold area corresponded to the Topcon, which subjectively showed a better defined mask too (Figure 1).

**Figure 2:**
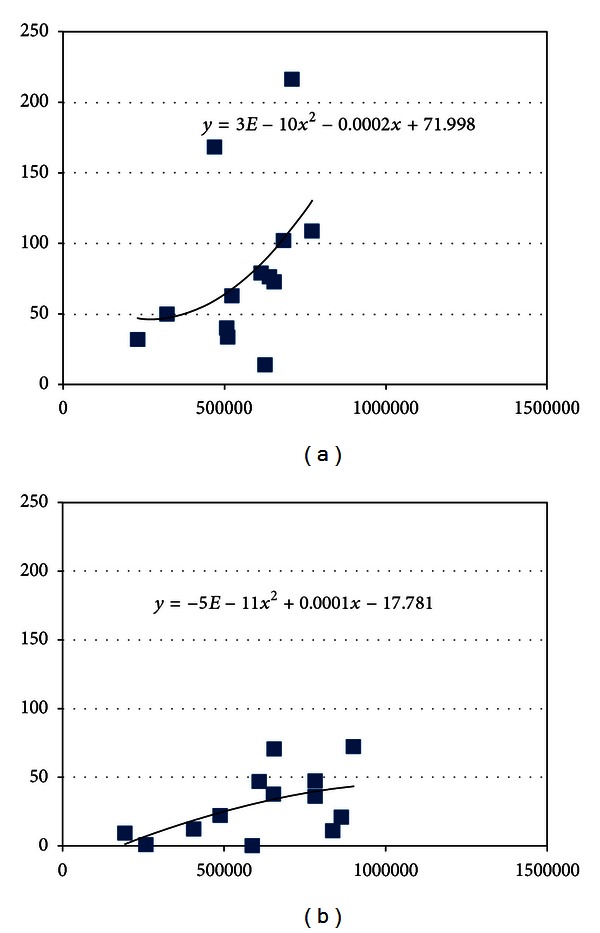
Correlation between the contrast (*D* value) in the *y*-axis and the area over the veins threshold (pixels) in the *x*-axis, for the Carl Zeiss (a) and the Topcon camera (b).

**Figure 3:**
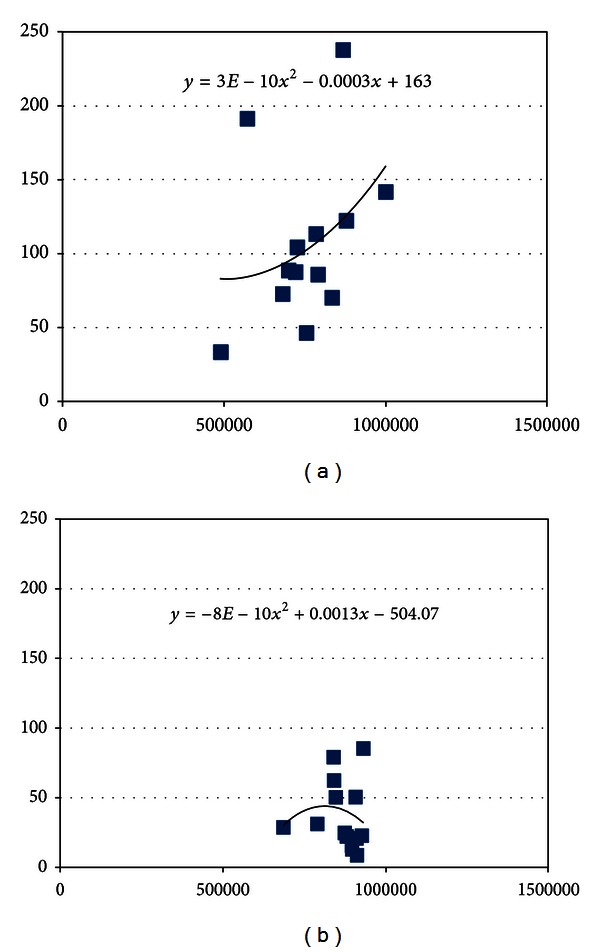
Correlation between the contrast (*D* value) in the *y*-axis and the area over the peripapillary ring threshold (pixels) in the *x*-axis, for the Carl Zeiss (a) and the Topcon camera (b).

These findings made us consider a possible correlation between the Δ values for contrast and the suprathreshold areas found in the autofluorescence photographs. The correlation graphs appeared to follow 2nd order polinomic equations, as showed in Figures 2 and 3.

For the brighter area over the veins threshold, we found no difference between the two cameras (*t* = 0.759, *P* value >0.05). We did find difference for the brighter area over the peripapillary ring threshold between the two cameras (*t* = 2.60 > 2.064, *P* < 0.05). The largest suprathreshold area corresponded to the Topcon. The Topcon camera subjectively showed a better defined mask (Figure 1).

These findings made us consider a possible correlation between the “Δ” values for contrast and the suprathreshold areas found in the autofluorescence photographs. The correlation graphs appeared to follow the 2nd order polinomic equations, as showed in Figures 2 and 3.

## 4. Discussion

The contrast value measured with the J Image software is an objective form to estimate the ability of a camera, to accurately capture an image, by reproducing details within the brightest and the darkest sides of a grayscale at the same time [13, 14].

We found that the peripapillary ring is closer to an absolute black value within the grayscale, and we think it is probably because the veins are narrow and closer to the brightest area around the fovea.

The differences in contrast values could be due to the illumination, reflectivity, and sensor capability. These factors can be influenced by the quality of the optical system, and of the sensor *per se*, but also by the software of any camera [15]. The contrast or dynamic range refers to the amount of grayscale in an image. For digital imaging, this describes the system's ability to reproduce tonal information by the difference between the lightest light and darkest dark of an image [16].

In the graphics of contrast versus suprathreshold area, we noticed a similar behavior of the curves for the two cameras. Nevertheless, the Microsoft Excel software estimated somehow similar equations for the Carl Zeiss. Both curves followed a polinomic behavior, where the suprathreshold area increased with the increase in contrast. Unlike the Carl Zeiss, the Topcon curves showed increasing contrast with the increase in the suprathreshold area, until contrast values (“Δ”) of around 45, where they showed a crest with further decline in the contrast as the area continued to increase. These findings suggest that the image quality worsen progressively at this point, even though the suprathreshold area continued to increase. The mask was better defined with the Topcon camera in all points.

## 5. Conclusions

The Carl Zeiss camera showed higher contrast values over the Topcon for autofluorescence imaging. This could be due to its Kodak Megaplus sensor 1.6, its illumination, optical system, quality of the filters, or capability of the software used. The precise reason is beyond the objective of our study.

Our study established objective parameters as cornerstones for the quantification of the autofluorescence imaging with eye fundus cameras. These parameters could also be used to objectively measure and determine changes in areas of hyper- or hypofluorescence and for their followup along time, every time a normal variability has been defined with a larger series of photographs to a given eye in a precise moment.
